# Building a Graph Signal Processing Model Using Dynamic Time Warping for Load Disaggregation

**DOI:** 10.3390/s20226628

**Published:** 2020-11-19

**Authors:** Kanghang He, Vladimir Stankovic, Lina Stankovic

**Affiliations:** Department of Electronic and Electrical Engineering, University of Strathclyde, Glasgow G1 1XW, UK; hekanghang@gmail.com

**Keywords:** NILM, graph signal processing, energy efficiency, load disaggregation

## Abstract

Building on recent unsupervised Non-intrusive load monitoring (NILM) algorithms that use graph Laplacian regularization (GLR) and achieve state-of-the-art performance, in this paper, we propose a novel unsupervised approach to design an underlying graph to model the correlation within time-series smart meter measurements. We propose a variable-length data segmentation approach to extract potential events, assign all measurements associated with an identified event to each graph node, employ dynamic time warping to define the adjacency matrix of the graph, and propose a robust cluster labeling approach. Our simulation results on four different datasets show up to 10% improvement in classification performance over competing approaches.

## 1. Introduction

The ongoing smart metering deployments worldwide [1,2] aim to maximize benefits of the smart grid by providing household aggregate energy consumption in real time, facilitating accurate billing, demand response [3], improved network maintenance, fault detection [4,5], and improved energy efficiency feedback.

Ambitious energy efficiency goals and the availability of smart meter data has re-ignited research on Non-Intrusive Load Monitoring (NILM) [6], i.e., identifying individual loads from the aggregated measurements (e.g., voltage, current, and active and reactive power) of smart meters [7,8,9]. However, despite the fact that the NILM problem has been researched for four decades [6], it remains a challenge, especially at sampling rates in the order of seconds and minutes, typical for wide-spread smart meters. Indeed, sensor noise from measurements, multiple appliances working at the same time, unknown appliances, multi-state appliances, base-load, and load fluctuation all act as “disaggregation noise” [10].

An appliance operation can be modeled as a finite state machine and then disaggregation can be performed based on a state transition model learnt during training [6]. The most popular examples of this are state-based NILM methods, including various Hidden Markov Model (HMM)-based NILM methods and their extensions [6,8,11,12,13]. Event-based NILM methods, on the other hand, are based on detecting the event of an appliance being switched on or off, or transiting from one operation state to another (e.g., in the case of washing machine). Event-based NILM methods usually share the following three steps: (1) Event detection: Detect time instance of the state change in the time-series aggregate data. (2) Feature construction and extraction: Compute and extract the electrical features, for example, the active power, from the detected event duration. (3) Classification and pattern matching: Classify the events into pre-defined labels (i.e., appliances) based on the features extracted.

For the final step, various classification methods have been used, including support vector machines (SVM) [14], neural networks [15,16], non-negative tensor factorization [17], k-means [18], decision trees (DT) [19], optimization-based methods [20], and Graph Signal Processing (GSP) [7].

Based on the inference method applied, NILM algorithms can be supervised, semi-supervised or unsupervised. In supervised NILM (e.g., [14,15,19]), a labeled dataset (e.g., a diary of appliance usage or plug-based appliance monitors) is used to train machine learning models, which are then used to disaggregate unseen, test data. Semi-supervised NILM methods (e.g., [7]) use a small amount of labeled data and many unlabeled samples to train the models. Finally, unsupervised methods look for unknown patterns, e.g., using a distance metric, and hence do not use any labeled data for training.

Deep learning approaches have become state of the art for supervised NILM, based on various architectures, including long short-term memory (e.g., [21]), auto-encoder (e.g., [22]), and convolutional neural networks (e.g., [23]). All these methods require a large amount of training data to attain good performance. Promising, transfer learning deep-learning-based methods have emerged (e.g., [16]), but they rely highly on similarity of appliances across different datasets.

Providing a large and accurate dataset for supervised learning is impractical due to the requirement for plug-level monitors or cumbersome time-diaries. Hence, unsupervised methods are attractive. Popular unsupervised NILM methods are based on Dynamic Bayesian network models and specifically Hidden Markov Models (HMM) and their extensions including Factorial HMM, Additive Factorial HMM, and Gaussian Latent Dirichlet Allocation [6,8,11,12,13,24]. However, HMM-based NILM methods require good quality data to build the models, suffer from noise caused by unknown appliances, and are ineffective for rarely used appliances or appliances that are never used alone [8,11,25]. Some other unsupervised methods include: time-series approaches ( e.g., [26]) that require building a database of time-series signatures necessary for pattern matching; fuzzy-clustering regression trees-based approaches [27,28]; various edge detectors, e.g., mean shift proposed in [29]; and optimization-based methods [30] that are limited to small number of appliances. Bonfigli et al. [31] provided an overview of unsupervised NILM methods with performance comparison.

All these methods are based on generating features from the detected edges and then clustering the extracted features. In contrast, the proposed work utilizes additional samples in the appliance signature, enhancing the extracted feature vectors and improving the inference.

GSP is an emerging approach that provides robust methods for signal filtering, denoising [32], clustering [25], and classification [7], where complex relationships between samples of high dimensional data are represented using graphs. The potential of GSP for the NILM problem was first demonstrated by Stankovic et al. [33] using Graph Laplacian regularization (GLR), followed by improved designs in the supervised approach of He et al. [7] and unsupervised approach of Zhao et al. [25], which currently represent the state of the art for low sampling rate measurements.

Although the GSP-based NILM designs in [7,25,33], and more recently in [9,34,35], and applied in [36,37,38], demonstrate potential, there are still limitations. In [7,25,33], GSP is used as a classification/ clustering tool using the change in power level when operation state changes (e.g., the change in aggregate power when an appliance is turned on or off). These methods rely only on a single power change and ignore fluctuations of power during the operation of the appliance. On the other hand, Kumar et al. [34] directly applied GSP classification on aggregated power measurements, but the approach showed high computation complexity (since each sample from aggregated measurements is related to one graph node) and poor performance when multiple appliances are operating at the same time.

To address the above limitations of current designs, in this paper, we propose a novel unsupervised method for NILM disaggregation using Dynamic Time Warping (DTW). The proposed approach segments aggregated power data into windows and associates them to graph nodes. Then, DTW distance, instead of Euclidean distance as used in [7,9,25,34,35,36,37], learns the underlying graph for clustering different data segments. Simulation results demonstrate significant performance improvement over the prior methods. Specifically, the key contributions are:

- a data segmentation approach to identify variable-length segments of potential events;
- DTW-based graph learning based on segmented data;
- a robust cluster labeling approach for efficient appliance labeling and post-processing; and
- an overall unsupervised NILM method comprising graphical signal pre-processing, data segmentation, graph design, and labeling that outperforms competing approaches.

The rest of this paper is organized as follows. Section 2 provides a background on disaggregation methods and GSP in NILM. Section 3 describes the proposed unsupervised approach with four steps listed as subsections. Section 4 introduces five benchmarks applied in this paper for the results comparison, which is presented in Section 5. The last section concludes the paper and highlights future work.

## 2. Background and Prior Work

In this section, we first introduce the notation and review relevant concepts of GSP. Then, we review prior work on GSP tools for NILM.

### 2.1. Notation and Background

All matrices are denoted by uppercase bold letters, such as X. Element in the *i*th row and *j*th column of the matrix X is denoted by $x_{i,j}$. Vectors are denoted by lowercase bold letter, such as x with the *i*th element denoted as $x_{i}$.

A vector of measurements x is represented by a graph, defined as G=(V,A), where V is the set of nodes and A is a weighted adjacency matrix. Each node $v_{i}\in V$ corresponds to an element $x_{i}\in x$. Each entry $a_{i,j}$ in A represents the weight of the edge between graph nodes $v_{i}$ and $v_{j}$, carrying information on similarity, i.e., correlation, between the elements $x_{i}$ and $x_{j}$. $a_{i,j}$ is usually defined by Gaussian Kernel weighting function:

(1) $$a_{i,j}=e^{\frac{-{\left(d\left(i,j\right)\right)}^{2}}{\sigma^{2}}},$$

where d(i,j) is the distance between $x_{i}$ and $x_{j}$, e.g., the Euclidean distance as in [7,9,25,34,35].

A combinatorial graph Laplacian matrix is defined as:

(2) L=D−A,

where D is a diagonal degree matrix and $d_{k,k}=\sum_{j=1}^{N}a_{j,k}$, where *N* is the length of x. Note that, since G is an undirected graph, A, as defined in (1), is symmetric ($a_{i,j}=a_{j,i}$) and real and L is a positive semi-definite matrix of dimension N×N.

Next, we define a graph signal y as a mapping from V to a set of complex numbers. For example, in the classification task, y is a set of classification labels representing classes to which the samples in x belong to; that is, $y_{i}\in y$ is the class of the sample $x_{i}\in x$.

GSP-based representation methods, including graph Laplacian regularization (GLR) and graph total variation (GTV) minimization, have been used in the past to classify time-series signals [32,39,40,41]. The main idea is to represent classification labels as a piece-wise smooth signal on graphs and then apply a graph signal smoothness prior. In particular, the classification labels, y, form a discrete graph signal that resides on G, where a classification label $y_{i}\in y$, corresponding to the observed feature vector $x_{i}$, indexes Vertex $v_{i}$.

Let *n* be the number of samples in the *N*-length vector x for which the class membership is known; that is, these *n* samples form a training set, and all classification labels $y_{i},i\leq n$ are known and fixed. All unknown labels to be determined, i>n, are initialized to zero. If the graph is designed such that vertices that correspond to observations with similar characteristics (and are consequently in the same class) are connected with high-value weighted edges, then the graph signal y is expected to change slowly from vertex to vertex, that is, it is piece-wise smooth.

Then, one can restore unclassified labels ($y_{i},i>n$) by finding the smoothest graph signal that minimizes the graph global smoothness, by minimizing GTV, defined as:

(3) $$TV_{G}\left(y\right)=\frac{1}{{\left||y|\right|}_{2}^{2}}||y-\frac{1}{|\lambda_{max}|}\mathrm{Ay}{\left|\right|}_{2}^{2},$$

where $\lambda_{max}$ is the largest-magnitude eigenvalue of A, or the graph global smoothness, often referred to GLR, whose quadratic form is given by:

(4) $$TV_{L}\left(y\right)=y^{⊤}\mathrm{Ly}=\sum_{i,j}{\left(y_{i}-y_{j}\right)}^{2}a_{i,j}.$$

Since, for undirected graphs, Laplacian representation is often desirable, due to many useful properties of graph Laplacian matrix [42,43], in this paper, we use GLR minimization.

Note that GLR minimization leads to real-valued solutions that are then, for binary classification, ‘quantized’ so that y∈{0,1}, where $y_{i}=1$ means that $x_{i}$ belongs to Class 1. Multi-class classification can then be performed as one-against-all approach for each class, one at a time. The method described above provides a powerful, scalable, and flexible data mining and signal processing approach, particularly suited for classification tasks when training periods are short and insufficient to build comprehensive class models [40,41].

### 2.2. Related Work

GSP-based methods have recently been proposed for tackling the event-based load disaggregation problem [7,9,20,25,34,35,36,37]. In [7,25], after the events (i.e., load state changes) are detected by thresholding, the feature of each event is represented using a power edge, that is, the change of aggregated power value when the detected event started or ended. This way, each event is associated with one graph node, and classified by minimizing Equation (4) [7]. Binary classifiers are run multiple times, once for each appliance. Zhao et al. [25] first applied GLR minimization to identify all events that belong to the cluster of a particular event type/class. Then, clustered events are removed and the procedure is repeated until all events are assigned to a cluster. Zhao et al. [37] provided a GSP-filter based pre-processing and graph-based edge matching post-processing to further improve the performance of event-based NILM methods. In [34,35], unlike classification/clustering labels defined as graph signal in [7,25], the authors directly used active power or power edges as graph signals.

The main issue of the above approaches [7,25,35,36] is the fact that they heavily depend on successful edge detection to isolate the power change events, and thus assign a single graph node to each detected event. However, due to the noisy nature of true aggregated data where many unknown loads are present, edge detection often fails. For example, if a true event is missed (i.e., a true negative) or false event is detected (i.e., false positive), the GSP classifier will fail. This situation is, to a certain extent, mitigated in [9], where a semi-supervised approach of high complexity is proposed, relying on time embedding, graph sparsification with loopy belief propagation and finally GLR or manifold regularization for classification.

To mitigate the effect of event detection failure, while keeping the complexity low, in this paper, we propose a novel graph generation approach for NILM, where each graph node is associated with a continuous time-series signal of power measurements, constituting the same steady-state, instead of an event sample. Each graph node is associated with a feature vector comprising power values and time information. Instead of Euclidean distance, used in the prior work [7,9,25,34,35,36], and due to the variable length of event segments, distance based on dynamic time warping (DTW) is used to estimate the level of correlation between segments, and then construct the graph. DTW is used for NILM in [19,26] to compare the distance between appliance “templates” and extracted segments. However, the approach of Liao et al. [19] requires a database that contains good quality templates.

In a related paper [44], GSP-based principal components analysis (PCA) is applied to microseismic data analysis. Then, the graph weight is calculated based on the sum of Euclidean distances. This scheme is included as benchmark.

## 3. Methodology

The main limitation of the prior GSP-based NILM approaches reviewed in the previous section, is the fact that they either ignore fluctuation of power values within the identified event, that is, rely only one value when the appliance is switching on/off or changing state [7,25], or suffer from high complexity [9,34,35,36]. This is in contrast to state-based approaches that track the changes of measured power, but are difficult to train [8,11,12]. To address these limitations, we propose an improved GSP-based NILM design, described next.

The proposed NILM-based system comprises a pre-processing block, data segmentation, clustering, and finally cluster labeling. Each of these blocks is described in the following subsections. We use $P_{i}$ to denote the active power measurement at time instant *i*, and $\Delta P_{i}=P_{i}-P_{i-1}$, for i>1.

### 3.1. Pre-Processing

The smart meter measurements are accompanied by unavoidable measurement noise. Furthermore, transient power values act as noise in the steady-state analysis. Pre-processing has been commonly used to reduce these effects (see [25] and references therein). It is especially important in the proposed scheme, as it relies on all collected measurements, instead of just an “edge”—i.e., the value of significant power change corresponding to appliance state changes, $\Delta P_{i}=P_{i}-P_{i-1}$.

We propose a two-stage pre-processing approach: firstly, median filtering for sharpening edges and improving data segmentation (discussed in the next subsection), and, secondly, bilateral filtering for denoising the signal and removing signal fluctuations to improve the clustering step (Section 3.3).

We sharpen signal edges via median filtering for $\Delta P_{i}$ to stand out more clearly. That is, if $|\Delta P_{i}|>Thr$, then the *i*th sample potentially belongs to the start/end of an event, and therefore an appliance state change. We update such samples using:

(5) $$\Delta P_{i}=\sum_{j=i-k}^{j=i+k}\Delta P_{j},$$

where *k* is an averaging window.

Next, graph-based bilateral (GB) filtering, as in [37], smooths signal fluctuations. For a vector signal x, with $x_{i}=\Delta P_{i}$, we design the underlying graph by setting the adjacency matrix A using Equation (1), based on Gaussian kernel function with σ being the scaling factor set heuristically:

(6) $$a_{i,j}=e^{\frac{-{\left(x_{i}-x_{j}\right)}^{2}}{\sigma^{2}}}.$$

Then, the diagonal degree matrix D is $d_{k,k}=\sum_{j=1}^{N}a_{j,k}$.

From the bilateral filter operator $D^{-1}A$, we obtain an optimization problem to restore the filtered, clean signal as:

(7) $$\min_{\hat{x}}\frac{1}{2}{\left|\right|\hat{x}-x\left|\right|}_{2}^{2}+\alpha \frac{1}{2}{\left|\right|\hat{x}-D^{-1}A\hat{x}\left|\right|}_{2}^{2},$$

where α is a parameter that trades-off the two terms. Equation (7) can be solved by calculating the first derivative of the cost function with respect to the filter input, and the closed form solution is [32]:

(8) $${\hat{x}}^{*}={\left(I+\alpha \left(I-D^{-1}A\right)*\left(I-D^{-1}A\right)\right)}^{-1}x,$$

where ${\hat{x}}^{*}$ is the output signal and I is the unit matrix. GB filtering is applied as in Equation (8) where x is a vector of consecutive $\Delta P_{i}$, given by Equation (5). As in [37], we set α to 1, which was experimentally determined to provide best results for the NILM problem at low sampling rates.

The performance gain due to pre-processing is experimentally evaluated in Section 5.3.

### 3.2. Signal Segmentation

In the following, we refer to $p_{i}$ and $\Delta p_{i}$ as filtered aggregate power and filtered power change value, respectively. Once the measured power signal is denoised and sharpened, the next step is to segment the signal and associate each segment to one graph node.

The idea is to group aggregate power samples if they belong to the same steady state. Data segmentation is achieved by comparing the value of the aggregate power change $|\Delta p_{i}|=p_{i+1}-p_{i}$ with a preset threshold Thrs. If $|\Delta p_{i}|>Thrs$, $p_{i+1}$ will be the first sample of a new segment. Otherwise, $p_{i}$ belongs to the same segment as the previous sample.

We use S to represent the set of all segments and $S_{i}$ is the *i*th segment, consisting of continuous samples of aggregated power corresponding to the *i*th estimated steady state. The preset threshold Thrs should be larger than the envisaged maximum variation during the same steady state but smaller than the minimum value of the state changes.

The proposed segmentation approach is given in Algorithm 1. Starting from i=1 and j=1, the algorithm assigns $p_{i}$ to $S_{j}$ and compares $|\Delta p_{i}|$ with Thrs. $p_{i+1}$ is assigned to $S_{j}$ if $|\Delta p_{i}|<Thrs$ or it is assigned to $S_{j+1}$ otherwise. If $p_{i+1}$ is assigned to $S_{j+1}$, we increase *j* by one. Then, we increment *i* and repeat the above steps until all samples from the aggregate power measurements p are assigned to one segment.

**Algorithm 1:** Data segmentation.

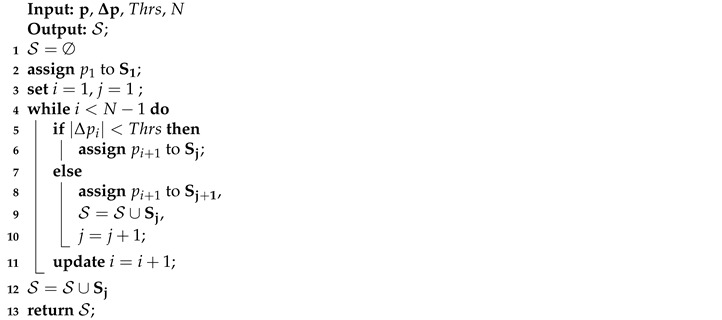



After the signal is segmented, clustering is performed to assign the segments to different clusters, as explained next.

### 3.3. GSP-Based Clustering

In this section, we introduce three unsupervised GSP (UGSP) clustering methods that differ in the way segments are defined and consequently the way the underlying graph is designed. The first approach segments the data and then resorts to DTW to build a graph. The other two methods do not use the segmentation approach described in Section 3.2, but instead rely on comparing fixed-length segments. Following the notation introduced in Section 2, a graph G=(V,A) is defined by graph nodes V and adjacency matrix A.

#### 3.3.1. UGSP-DTW Method

Each node $v_{i}\in V$ is assigned to a segment $S_{i}\in S$ obtained as described in Section 3.2. Since $S_{i}$ and $S_{j}$ are vectors of different lengths, we use DTW distance to measure their similarity. DTW is commonly used for measuring similarity between two sequences [19]. Thus, we design the graph adjacency matrix as:

(9) $$a_{i,j}=e^{\frac{-DTWdist{\left(S_{i},S_{j}\right)}^{2}}{\sigma^{2}}},$$

where σ is the scaling factor, iteratively tuned as in [45] based on graph Fourier transform, and DTWdist(a,b) is the distance between sequences a and b.

DTW is a commonly used metric for measuring similarity between two sequences of possibly different lengths. It is very popular in speech recognition and lately data mining. Given two signals of possibly different lengths, DTW performs a non-linear mapping of one signal to another by minimizing the distance between the two signals by finding an optimal mapping path via dynamic programming. In particular, the DTW distance between two sequences $p=[p_{1},\ldots ,p_{n}]$ and $q=[q_{1},\ldots ,q_{m}]$ is calculated as follows.

As part of initialization, for all i,j>0,D(i,0)=D(0,j)=∞ and D(0,0)=0. As a boundary constraints, the mapping path starts at (1,1) and ends at (n,m). That is, let

$$d\left(i,j\right)=\sqrt{{\left(p_{i}-q_{j}\right)}^{2}}$$

be a distance between points $p_{i}$ and $q_{j}$, where 1≤i≤n and 1≤j≤m. Then, D(i,j) is accumulated DTW distance between vectors $\left[p_{1}\ldots p_{i}\right]$ and $\left[q_{1}\ldots q_{j}\right]$ given by

D(i,j)=d(i,j)+min{D(i−1,j),D(i−1,j−1),D(i,j−1)},

and D(n,m) is the final DTW distance between p and q. More details can be found in [46].

Similar to He et al. [7], we define the graph signal s as the classification label of each data segment $S_{i}$. Starting from the first sample of the graph signal s, we adopt binary GSP-based classification to find all samples $s_{i}$, where i>1, that belong to the same cluster as $s_{1}$. That is, first we set $s_{1}=1$, and all remaining samples of graph signals are set to zero. Then, GLR is adopted to estimate a graph signal with minimum variation with respect to the underlying graph. The optimization problem is:

(10) $$s^{*}=\min_{s}\left|\right|s^{⊤}\mathrm{Ls}\left|\right|,$$

where L is a combinatorial Laplacian matrix of the graph as defined in Equation (2).

The closed-form solution to the optimization problem in (10) is [47,48]:

(11) $$s^{*}=-L_{2:N+1,2:N+1}^{\#}s_{1}L_{1,2:N+1}^{⊤},$$

where $L_{2:N+1,2:N+1}^{\#}$ is the pseudo-inverse of $L_{2:N+1,2:N+1}$. Note that, for a matrix L, the subscript $x_{1}:x_{2},y_{1}:y_{2}$ is a sub-matrix that contains columns $x_{1}$ to $x_{2}$ and rows $y_{1}$ to $y_{2}$ of L. If $s_{i}^{*}>T_{s}$, we set $s_{i}=1$, i.e., we assign it to the same cluster as the starting sample; otherwise, $s_{i}$ is set to zero. Similar to He et al. [7], we set $T_{s}=0.5$. Then, we remove all clustered segments, including the first one, and repeat the above steps until all segments are assigned to a cluster.

#### 3.3.2. UGSP_PCA-FIX and UGSP_GPCA-FIX

An alternative approach is to reduce dimensionality of the segments leading to shorter fixed-length segments and estimate correlation of the newly generated segments using Euclidean distance. Principal component analysis (PCA) is a commonly used statistical procedure that uses an orthogonal transformation to convert a set of possibly correlated variables into a set of linearly uncorrelated variables called principal components [49] and maximize the sample variance. After data segmentation (Section 3.2), PCA is performed on each segment of samples to obtain the principle components. Since components of all segments are of the same length, the graph adjacency matrix is defined using the sum of Euclidean distances, akin to Equation (6), by replacing DTW distance with Euclidean distance in Equation (9). The remaining clustering and labeling steps are the same as in the proposed scheme.

We also provide results for Graph Principal Component Analysis (GPCA) inspired by Taylor et al. [44]. In GPCA, for each data segment, we build a graph G=(V,A), where each graph node $v_{i}\in V$ is associated with a sample inside the data segment. The adjacency matrix A and the graph Laplacian matrix L are defined using Equation (1) with Euclidean distance measure and Equation (2), respectively. As with PCA, GPCA finds the new coordinates (i.e., principal components) of each data segment. The solution to the generalized eigenvalue decomposition problem shown in (12) provides the new parameterized data with the same dimension [44,50].

(12) $$\left(D-A\right)\psi_{k}=\lambda_{k}D\psi_{k},$$

where $\psi_{k}$ and $\lambda_{k}$ are the *k*th eigenvector and eigenvalue of the graph Laplacian matrix, respectively. This is similar to the traditional PCA, but has an advantage that provides a more general model of the raw data. The GPCA method shares the same remaining steps as the PCA method.

#### 3.3.3. UGSP-FIX and UGSP_OPT-FIX Methods

The segmentation approach proposed in Algorithm 1 relies on a manually set threshold Thrs. To avoid that, UGSP-FIX and UGSP_OPT-FIX methods use fixed-length segments of duration *T* samples. Then, we define the adjacency matrix A:

(13) $$a_{i,j}=e^{\frac{-\sum_{t=1}^{T}\omega_{t}{\left(p_{i+t-l}-p_{j+t-l}\right)}^{2}}{\sigma^{2}}},$$

where $l=\frac{T+1}{2}$, *T*, the length of the segment, is assumed to be an odd number, and σ is the scaling factor, tuned as in [45]. $\omega_{t}$, with $\sum_{t=1}^{T}\omega_{t}=1$, is the weight of the *t*th sample, enabling the ability to assign different levels of importance to different segments. Note that $a_{i,j}$ is the averaged weighted sample-by-sample Euclidean distance between Segments *i* and *j*. In the low-complexity UGSP-FIX method, we set $\omega_{t}=1$ for $t=\frac{T+1}{2}$, and zero otherwise. In UGSP_OPT-FIX method, we optimize $\omega_{t}$’s via a full-search algorithm for t=1,…,T [51], which is possible since *T* is kept small.

The remaining GSP-based classification steps are the same as in Method UGSP-DTW.

### 3.4. Cluster Labeling

After all data segments are grouped into clusters, the clusters need to be assigned to correct labels, that is, appliances or disaggregated loads. For each cluster $C_{m}$, we first calculate the average length $\bar{H_{m}}$ of all segments within the cluster. If $\bar{H_{m}}$ is less than $Thr_{H}$, we set the corresponding cluster $C_{m}$ as noise, belonging to non-classified segments, $L_{1}$. $Thr_{H}$ is a heuristically set threshold to identify noisy clusters with short data segments.

Next, we compare $\bar{P_{C_{m}}}$, the mean of active power values of all measurements within cluster $C_{m}$, with $Thr_{l}$ and $Thr_{h}$, which are the lower and upper bound thresholds. Clusters with $\bar{P_{C_{m}}}$ larger than $Thr_{h}$ or $\bar{P_{C_{m}}}$ less than $Thr_{l}$ are also labeled as $L_{1}$. For the remaining clusters, $\bar{P_{C_{m}}}$ is compared with $\bar{P_{n}^{l}}$ for n∈[1,K], where *K* is the total number of labels, and for each preset label $L_{n}$, $\bar{P_{n}^{l}}$ is the expected power value of the appliance labeled as $L_{n}$, which can be estimated using manufacturer information or expert knowledge. Label $L_{n},n=1,\ldots ,K$ denotes the case when individual appliances is in the ON-state with no other appliances in the ON-state ($\bar{P_{n}^{l}}$ is the estimated expected wattage of an appliance), or a group of appliances is in the ON-state simultaneously ($\bar{P_{n}^{l}}$ is the sum of the estimated expected wattage of a group of appliances). For multi-state appliances, each state is treated as an individual appliance in the ON-state.

Cluster *m* is assigned to the label $L_{n}$ if $|\bar{P_{C_{m}}}-\bar{P_{n}^{l}}|$ is the minimum over all n∈[1,K]. Clusters with $|\bar{P_{C_{m}}}-\bar{P_{n}^{l}}|$ larger than the preset threshold $Thr_{d}$ will be assigned to an unknown appliance which is also labeled as $L_{1}$. The pseudocode of the cluster labeling method is shown in Algorithm 2, where C is the set of all clusters. 

**Algorithm 2:** Cluster labeling.

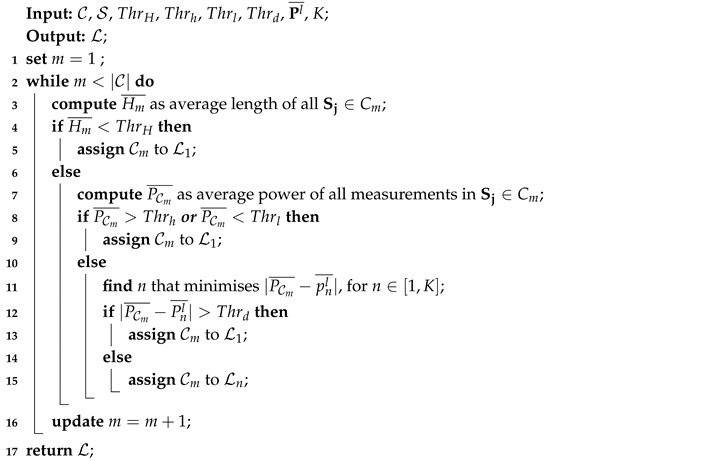



After all clusters are assigned a label, the coherence of multi-state appliances is considered to further refine the labeling results. Any cluster with only a single observed state that was assigned to a multi-state appliance label, which cannot appear alone, is assigned to the second best single-state label, only if in Step 11 of Algorithm 2 the following condition is satisfied: $|\bar{P_{C_{m}}}-\bar{P_{l}^{r}}|$ is the second smallest and is within threshold $Thr_{d}$. The average duration $\bar{H_{m}}$ of cluster $C_{m}$ is also used as additional information to help improve the labeling accuracy. If $\bar{H_{m}}$ is much longer (beyond a pre-defined time threshold) than the expected working duration of the corresponding appliance, the second minimal difference in Step 11 is used to correct the label.

The parameters of the proposed method are set as following: $Thr_{H}=5$ is a heuristically set threshold to avoid picking up too-short clusters due to noise. $Thr_{d}=200$ W is the maximum difference allowed between the average power of a cluster and the expectation of labels. $Thr_{l}$ is slightly larger (by 10 W) than the base-load. $Thr_{h}$ is set to the double of the maximum expected power of any appliance. The parameters are set to trade off performance and complexity. Changing these parameters by 10–20% will not influence the accuracy of the results.

## 4. Benchmarks

This section briefly describes three benchmarks. The first scheme is natural extension of the proposed UGSP-DTW scheme to the semi-supervised learning setting. The second scheme uses an alternative clustering approach. The final scheme [37] shares the same GSP-based clustering method but a different segmentation approach, and is shown in [37] to be superior to state-of-the-art state-based and event-based unsupervised approaches.

### 4.1. Semi-Supervised GSP

A semi-supervised GSP approach, SGSP-DTW, shares the same pre-processing and data segmentation steps as the proposed UGSP-DTW method. After data segmentation (Section 3.2), graph-based binary classification, similar to He et al. [7], is performed, instead of clustering (see Section 3.3) with the adjacency matrix given by (9).

### 4.2. DBSCAN

Density-based spatial clustering of applications with noise (DBSCAN) is a commonly used data clustering algorithm which does not require the number of clusters to be specified. DBSCAN is usually used for spatial point set clustering. As in [45], DBSCAN is performed instead of GSP-based clustering as a benchmark. Graph nodes are regarded as points in the point set data, and DTW distance is used to represent the distance between the nodes. Note that DBSCAN relies heavily on two parameters that need to be pre-set, the minimum number of neighboring samples needed, and the minimum number of samples in a cluster. As confirmed by the simulation results presented in the next section, DBSCAN is ineffective for appliances with low frequency of usage.

We also include a comparison with the best unsupervised methods reported in [52], where DBSCAN is evaluated with a Euclidean distance measure. This helps us further understand the importance of the DTW distance metric. Furthermore, in [52], several unsupervised NILM pre-processing methods are evaluated with DBSCAN clustering, including median filtering, edge sharpening, graph bilateral filtering and their various combinations. We denote this method as DBSCAN-Euclidean method [52] and report the results only for the appliances and datasets considered in [52].

### 4.3. Event-Based GSP

We also compare the proposed method with the event-based unsupervised GSP-based NILM of Zhao et al. [25], but apply the pre-processing steps of Section 3.1 on the raw data.

## 5. Results and Discussion

In this section, we present our experimental results. First, in Section 5.2, we introduce performance measures and datasets. Section 5.3 presents the performance of pre-processing. The following subsection compares the five segmentation and clustering methods introduced in Section 3.3. The last subsection compares the proposed DTW-based unsupervised GSP method with the benchmarks described in Section 4.

### 5.1. Datasets

We used two open-source datasets: REDD dataset that contains measurements at 1 Hz sampling rate [53] and REFIT dataset at 1/8 Hz sampling rate [54]. The REDD dataset contains data from US houses and is widely used for evaluation of various NILM approaches, containing few unknown appliances and relatively low “disaggregation noise” [10]. The REFIT dataset is more realistic, containing continuous recording of aggregate and submetered appliances over two years from UK houses as well as many unknown appliances and therefore a high level of “disaggregation noise” [10].

Both datasets contain a single time-series aggregate power signal (measured in Watts [W]). We used two houses from each dataset, selected to include typical appliances and various noise levels [10]. Appliance-level plug readings were used to determine ground truth.

In the results tables, we use BGFI to label bathroom GFI, DW dishwasher, R refrigerator, KO kitchen outlet, MW microwave, WD wash dryer, S stove, F-F fridge freezer, WM washing machine, T toaster, and K kettle. We note that, besides these appliances, each house contains a number of unknown appliances that are considered to be noise. Generally, REFIT houses are “noisier”, in the sense of containing many unknown appliances. All results are provided for one month worth of data.

### 5.2. Performance Metrics

We used F-measure ($F_{M}$) over all samples to evaluate NILM classification performance defined as: PR=TP/(TP+FP), RE=TP/(TP+FN), $F_{M}=2*\left(PR*RE\right)/\left(PR+RE\right),$ where TP (true positive) is the number of samples which the NILM method correctly decided that the appliance was on, FP (false positive) is when the NILM estimates the appliance is on while the appliance is actually off, and FN (false negative) considers samples that are detected as off by the NILM algorithm while the appliance is in fact on. PR (precision) captures the correctness of detection—the higher is the PR, the fewer are the FPs. On the other hand, high RE (recall) means a low number of FNs, which implies that a higher percentage of appliance on states are detected correctly. $F_{M}\in \left[0,1\right]$ balances PR and RE.

### 5.3. Pre-Processing Gain Evaluation

The proposed pre-processing (Section 3.1) introduces additional complexity and delay. Hence, in this subsection, we compare the proposed schemes with and without pre-processing to evaluate the gain obtained via the proposed pre-fltering. We denote R-SGSP-DTW and R-UGSP-DTW (where R = raw measurements) to represent DTW-based semi-supervised and unsupervised GSP methods, respectively, without pre-processing applied on the raw measurements.

Table 1 shows the results for REFIT House 2. For both semi-supervised and the proposed unsupervised load disaggregation methods, pre-processing provides significant improvement of over 0.3, for all appliances. The gain is smallest for KO due to the relatively low noise levels for this appliance. Due to obvious performance gains of pre-processing, in the following section, we present the results of the proposed scheme and benchmarks, all with the pre-processing method of Section 3.1 (see [37] for a detailed study of various pre-processing methods for NILM, including graph bilateral filtering).

### 5.4. Comparison between Segmentation Methods

To quantify the gains from the proposed data segmentation, in this section, we compare the three proposed methods, UGSP-DTW, UGSP-FIX, UGSP_OPT-FIX, UGSP_PCA-FIX, and UGSP_GPCA-FIX, introduced in Section 3.3.

The parameters of the proposed method are set as follows: $Thr_{H}=5$ W is the threshold to isolate noise clusters with short data segments, since we observe that 5 W and lower generally represents appliances on stand-by, and not in active use, i.e., ON state; $Thr_{d}=200$ W is the maximum distance from the average power of a cluster to the state value to be associated with that cluster label; $Thr_{l}$ is set slightly over the estimated base-load; and $Thr_{h}$ is set to twice the maximum power of any appliance in the house.

Table 2 shows the F-measure results of the five segmentation approaches for House 1 in the REDD dataset. For the fixed-segment methods, T=7 to include six neighboring samples. First, when comparing UGSP_OPT-FIX and UGSP-FIX, we can see that optimizing weights provides negligible performance gain across all six appliances. Secondly, exploiting correlation and reducing dimensionality via PCA- and GPCA-based methods provides an additional performance gain over both fixed-segmentation methods. However, PCA- or GPCA-based methods often do not capture well the structure of raw data leading to a large difference between two data segments with the same label. Finally, there is a noticeable and significant performance gain of the proposed variable-length segmentation UGSP-DTW over both fixed-segmentation methods and PCA- and GPCA-based methods, indicating that DTW preserves some key information in the segments which PCA and GCPA removes during the transformation.

### 5.5. Comparison against NILM Benchmarks

In this section, we evaluate the robustness of the proposed UGSP-DTW NILM method against the benchmark algorithms described in Section 4, namely SGSP-DTW ( DTW-based semi-supervised GSP method (Section 4.1)), UGSP_PCA-FIX (PCA-based unsupervised GSP method (Section 3.3.2)), UGSP_GPCA-FIX (GPCA-based unsupervised GSP method (Section 3.3.2)), DBSCAN-DTW (DTW-based DBSCAN method (Section 4.2)), and UGSP by Zhao et al. [25]. The results are given in Table 3 and Table 4, for two REDD houses, and Table 5 and Table 6 for two houses from the REFIT dataset.

Table 3, Table 4, Table 5 and Table 6 consistently show that the proposed UGSP-DTW method provides the best disaggregation accuracy for most appliances in all four houses. For the two REDD houses, which generally have lower disaggregation noise [10], the proposed method is superior to other methods for all appliances except R in House 2, when DBSCAN shows the best results. Note that, since refrigerator is always on, as expected, DBSCAN shows high performance. DBSCAN is a density-based clustering which is highly dependent on the size of each cluster and the minimum number of neighbor samples. Thus, for appliances, such as kettle, dishwasher, and microwave, with low frequency of use, DBSCAN is not effective, whereas, for the appliances such as refrigerator that are always on, DBSCAN shows high performance. We can also see that the best performing DBSCAN-Euclidean method of Khazaei et al. [52] performs generally worse than the DBSCAN-DTW-based approach, demonstrating the improvement due to the DTW distance measure.

While it may be expected that SGSP-DTW outperforms all unsupervised methods, the results in the tables show that this is generally the case for all unsupervised methods except for the proposed UGSP-DTW and DBSCAN-DTW methods. This is due to the post-processing cluster labeling step of the proposed Algorithm 2 in UGSP-DTW and DBSCAN-DTW. The unsupervised state-of-the-art methods of Zhao et al. [25] and Khazaei et al. [52], which rely on a fixed Euclidean distance, perform worse that the proposed method except for a slight improvement for DW in REFIT House 2.

Overall, DTW-based approaches outperform other methods, demonstrating the value of the proposed variable-length segmentation and DTW distance measure.

## 6. Conclusions and Future Work

In this paper, we propose a DTW-based unsupervised GSP method for NILM. The proposed method is based on pre-processing, segmenting smart meter data, and clustering the segments using GSP-based clustering. We demonstrate the value of variable-length segmentation methods over fixed-length and transformation-based segmentation, and the value of the proposed cluster labeling method through rigorous experimentation on four REDD and REFIT datasets and four state-of-the-art benchmarks.

Future work includes providing an efficient real-time implementation integrating the approach into smart home decision support systems for demand response as well as designing advanced energy feedback mechanisms. Another interesting area of research is assessing the quality of the NILM output without relying on ground-truth. Future work also includes enlarging the set of features used for power disaggregation. These features could be reactive power, voltage/current, and other measurements, such as date, time, and weather.

## Figures and Tables

**Table 1:** F-measure for two GSP-based methods with and without pre-processing applied to REFIT House 2.

	F-F	WM	DW	TV	MW	T	K
R-SGSP-DTW	0.53	0.47	0.34	0.04	0.37	0.48	0.77
SGSP-DTW	0.75	0.64	0.63	0.11	0.68	0.58	0.87
R-UGSP-DTW	0.52	0.44	0.23	0.01	0.45	0.33	0.81
UGSP-DTW	0.83	0.70	0.61	0.38	0.79	0.72	0.90

**Table 2:** Comparison of $F_{M}$ for the segmentation methods introduced in Section 3.3 for REDD House 1.

	BGFI	DW	R	KO	MW	WD
UGSP-FIX	0.33	0.41	0.37	0.39	0.45	0.24
UGSP_OPT-FIX	0.33	0.42	0.49	0.40	0.45	0.25
UGSP_PCA-FIX	0.45	0.62	0.61	0.55	0.67	0.61
UGSP_GPCA-FIX	0.55	0.69	0.70	0.61	0.72	0.63
UGSP-DTW	0.65	0.77	0.92	0.86	0.74	0.73

**Table 3:** Comparison of F-measure for REDD House 1.

	BGFI	DW	R	KO	MW	WD
UGSP-DTW	0.65	0.77	0.92	0.86	0.74	0.73
SGSP-DTW	0.53	0.67	0.89	0.81	0.73	0.66
DBSCAN-DTW	0.60	0.71	0.88	0.82	0.81	0.69
DBSCAN-Euclidean [52]	-	0.54	-	-	-	0.67
UGSP [25]	0.41	0.62	0.85	0.78	0.61	0.68

**Table 4:** Comparison of F-measure for REDD House 2.

	MW	WD	DW	R	S	KO
UGSP-DTW	0.94	0.85	0.75	0.88	0.83	0.78
SGSP-DTW	0.88	0.68	0.53	0.89	0.71	0.68
DBSCAN-DTW	0.81	0.68	0.69	0.92	0.70	0.63
UGSP_PCA-FIX	0.56	0.62	0.51	0.43	0.24	0.41
UGSP_GPCA-FIX	0.62	0.67	0.61	0.37	0.38	0.14
UGSP [25]	0.69	0.75	0.57	0.43	0.61	0.44

**Table 5:** Comparison of F-measure for REFIT House 2.

	F-F	WM	DW	TV	MW	T	K
UGSP-DTW	0.83	0.70	0.61	0.38	0.79	0.72	0.90
SGSP-DTW	0.75	0.64	0.63	0.11	0.68	0.58	0.87
DBSCAN-DTW	0.77	0.61	0.62	0.20	0.45	0.61	0.82
DBSCAN-Euclidean [52]	-	0.21	0.63	-	-	-	
UGSP_PCA-FIX	0.61	0.53	0.44	0.17	0.51	0.49	0.68
UGSP_GPCA-FIX	0.66	0.7	0.34	0.25	0.63	0.57	0.73
UGSP [25]	0.62	0.58	0.64	0.14	0.69	0.55	0.78

**Table 6:** Comparison of F-measure for REFIT House 6.

	F	K	T	DW	MW
UGSP-DTW	0.88	0.77	0.44	0.69	0.70
SGSP-DTW	0.81	0.79	0.45	0.57	0.63
DBSCAN-DTW	0.76	0.69	0.30	0.45	0.65
UGSP_PCA-FIX	0.72	0.67	0.39	0.58	0.54
UGSP_GPCA-FIX	0.75	0.66	0.34	0.52	0.59
UGSP [25]	0.46	0.74	0.37	0.43	0.57
